# Amino acid sensing in hypothalamic tanycytes via umami taste receptors

**DOI:** 10.1016/j.molmet.2017.08.015

**Published:** 2017-09-14

**Authors:** Greta Lazutkaite, Alice Soldà, Kristina Lossow, Wolfgang Meyerhof, Nicholas Dale

**Affiliations:** 1 School of Life Sciences, University of Warwick, Coventry, CV4 7AL, UK; 2 Department of Molecular Genetics, German Institute of Human Nutrition Potsdam-Rehbruecke, Arthur-Scheunert-Allee 114-116, 14558, Nuthetal, Germany

**Keywords:** Hypothalamic tanycytes, Taste receptors, Tas1r1/Tas1r3, mGluR4, Amino acids, Appetite

## Abstract

**Objective:**

Hypothalamic tanycytes are glial cells that line the wall of the third ventricle and contact the cerebrospinal fluid (CSF). While they are known to detect glucose in the CSF we now show that tanycytes also detect amino acids, important nutrients that signal satiety.

**Methods:**

Ca^2+^ imaging and ATP biosensing were used to detect tanycyte responses to l-amino acids. The downstream pathway of the responses was determined using ATP receptor antagonists and channel blockers. The receptors were characterized using mice lacking the *Tas1r1* gene, as well as an mGluR4 receptor antagonist.

**Results:**

Amino acids such as Arg, Lys, and Ala evoke Ca^2+^ signals in tanycytes and evoke the release of ATP via pannexin 1 and CalHM1, which amplifies the signal via a P2 receptor dependent mechanism. Tanycytes from mice lacking the *Tas1r1* gene had diminished responses to lysine and arginine but not alanine. Antagonists of mGluR4 greatly reduced the responses to alanine and lysine.

**Conclusion:**

Two receptors previously implicated in taste cells, the Tas1r1/Tas1r3 heterodimer and mGluR4, contribute to the detection of a range of amino acids by tanycytes in CSF.

## Highlights

• Hypothalamic tanycytes can detect amino acids in cerebrospinal fluid.
• The mechanism is taste receptor-dependent.
• Tas1r1/Tas1r3 mediates responses to l-arginine and l-lysine.
• mGluR4 mediates responses to l-alanine and partially those of l-lysine.
• ATP release from tanycytes evoked by amino acids reaches into the arcuate nucleus.

## Graphical abstract

**Figure:**
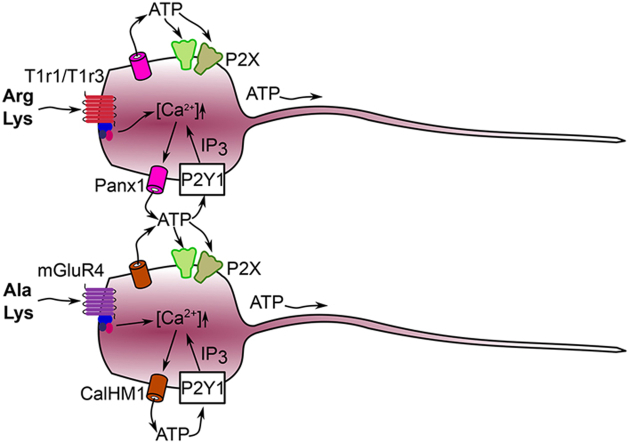

## 1 Introduction

Amino acids have been long known to be the most efficient type of nutrient at satisfying hunger and providing an extended period of satiety [1]. Some well understood reasons for this effect are slower digestion of protein-rich foods, as well as their ability to keep blood glucose levels relatively constant, thus reducing food cravings that can otherwise occur soon after a meal. However, it has become evident over the past few decades that the brain is a key player in energy homeostasis, and amino acids can have a satiating effect even when the digestive system is bypassed. Direct intracerebroventricular (ICV) injections of amino acids into the hypothalamus suppressed appetite in rats [2], [3]. Extensive work by Gietzen et al. has shown that the brain amino acid sensing mechanisms are so specific that an injection of one essential amino acid could reverse the adverse effect of a diet lacking that amino acid [4]. Further amino acid detection mechanisms have been described in the mediobasal hypothalamus, where an injection of leucine inhibited feeding by activating the mammalian target of rapamycin (mTOR) pathway [2], and in lateral hypothalamus, where hypocretin/orexin neurons, involved in feeding and sleep behaviors and speculated to link hypothalamic and cortical networks, have been shown to detect non-essential amino acids [5].

The hypothalamus receives other signals relevant to eating and food-related behaviors from the periphery as well as various areas in the brain and integrates the available information to provide an appropriate response to maintain energy homeostasis [6]. Peripheral food intake-related signals such as ghrelin, produced by an empty stomach [7], or leptin, secreted by the adipose tissue [8], access the hypothalamus via fenestrated capillaries around the third ventricle, and are transported by tanycytes, radial glia-like cells that line the wall of the ventricle, into the arcuate nucleus (ARC) [9], [10]. Ghrelin then activates the orexigenic neuropeptide Y-producing (NPY) neurons [11]. Leptin, on the other hand, inhibits NPY and agouti related peptide-producing (AgRP) neurons but excites anorexigenic proopiomelanocortin-producing (POMC) neurons [12].

One population of hypothalamic cells that is potentially a key player in energy homeostasis is the tanycytes. Their position in the wall of the third ventricle gives them privileged access to the ventricular cerebrospinal fluid (CSF); their long processes project into the nuclei that control energy homeostasis and come into close contact with the neurons of those nuclei, which suggests potential for communication between tanycytes and hypothalamic neurons [13]. Mouse tanycytes express ciliary neurotrophic factor (CNTF), the administration of which reduces food intake therefore counteracting positive energy balance in obese rodents [14]. Tanycytes have also been described as diet-responsive neural stem cells. Lineage tracing studies have demonstrated that tanycytes can generate new neurons and glia, meaning that the neuronal networks of the hypothalamus are highly plastic and can be remodeled by diet [15].

Tanycytes have long been hypothesized to be glucosensitive utilizing a mechanism similar to that of pancreatic beta cells, depending on specific glucose transporters, glucokinase and ATP-sensitive K^+^ channels (K_ATP_), which have been shown to be present in tanycytes [16]. Direct evidence shows that tanycyte glucosensing occurs via an ATP receptor-dependent mechanism [17], [18] and, in a large proportion of tanycytes, depends on the sweet taste receptor (Tas1r2/Tas1r3 heterodimer) [19]. Tas1r2/Tas1r3 is normally found in the taste cells of the tongue, surrounded by cells expressing receptors for other taste modalities [20]. A closely related receptor, the Tas1r1/Tas1r3 heterodimer, which shares a subunit with the sweet taste receptor, contributes to the perception of the umami taste (savory taste of glutamate in humans, or most non-aromatic l-amino acids in rodents) [21]. This suggested to us a novel hypothesis, that tanycytes could sense amino acids via this receptor. As amino acids are important signals of satiety, determination of whether they might detect amino acids would be an important advance in understanding the possible functions of these cells. The logic behind the experimental design of our study, the relevant controls, and the main results are summarized in Supplementary Table 1.

## 2 Methods

### 2.1 Brain slices

Male Sprague-Dawley rats (13–21 days old), male and female C57BL/6J mice (4–18 weeks old), and male and female *Tas1r1*-KO mice (5–15 weeks old, kindly provided by the Meyerhof lab) were humanely killed in accordance with the UK Animals (Scientific Procedures) Act 1986. The brain was rapidly dissected and placed in ice-cold artificial cerebrospinal fluid (aCSF: 124 mM NaCl, 26 mM NaHCO_3_, 1.25 mM NaH_2_PO_4_, 3 mM KCl, 2 mM CaCl_2_, 10 mM glucose; saturated with 95% O_2_/5% CO_2_; osmolarity 300 mOsm) with additional 10 mM magnesium. 300 μm (for Ca^2+^ imaging) and 400 μm (for ATP biosensing) thick coronal sections were cut in the area of interest using a Microm HM 650 V vibrating blade microtome. The slices containing the hypothalamus were cut in half down the midline to the third ventricle leaving its walls intact, and through the median eminence to expose the tanycyte layer. These were then put into aCSF at 34–37 °C to incubate for 30–45 min and later transferred to room temperature low-glucose (1 mM glucose + 9 mM sucrose to maintain osmolarity at 300 mOsm) aCSF, where they were maintained throughout the experiment.

Experiments in Results Sections 3.1, 3.2, 3.3 were performed on rat brain slices; data in 3.4 were collected from mice.

### 2.2 Drugs and drug application

**Figure 1:**
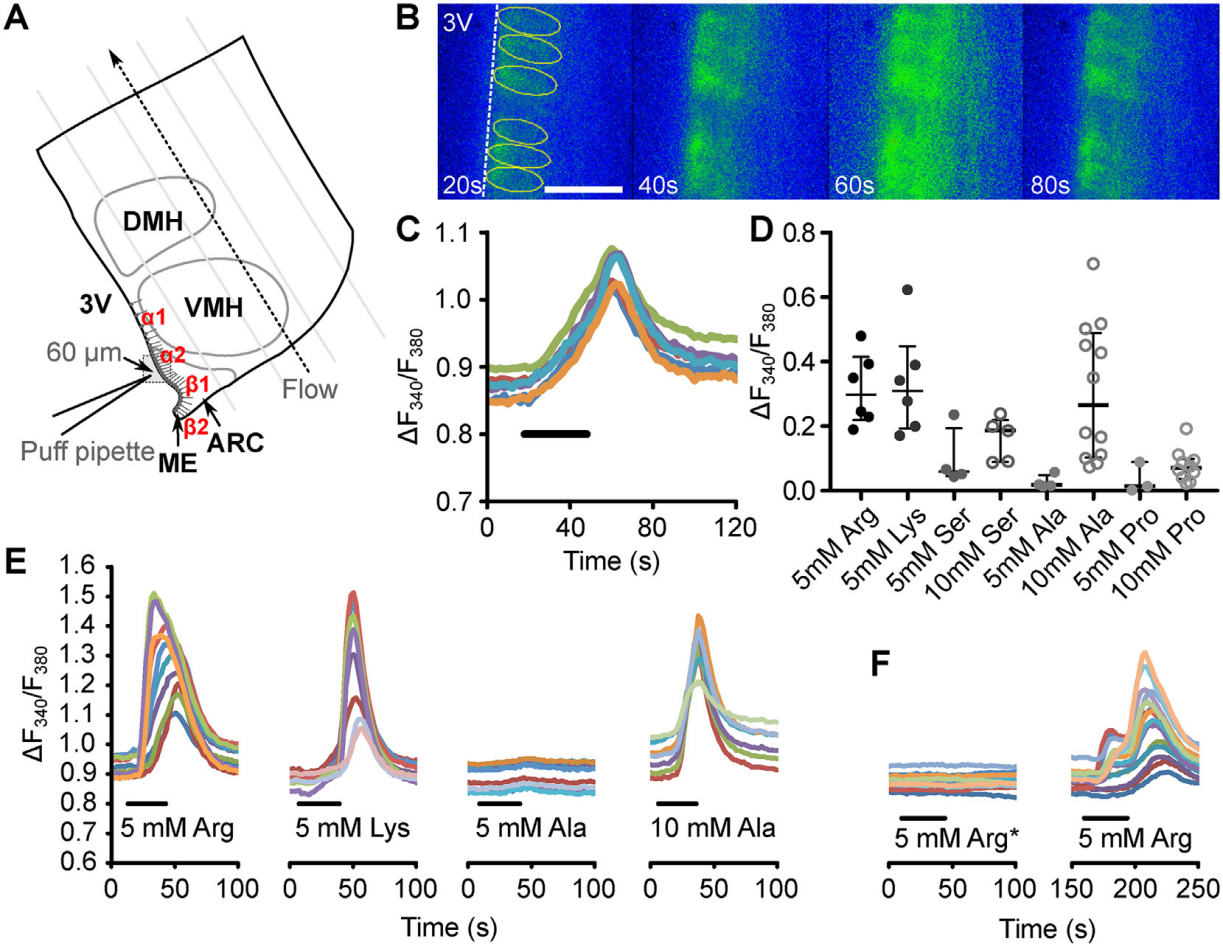
**Tanycytes respond to****l****-amino acids.** (**a**) The schematic graph shows the position of the brain slice and the puffing pipette within the imaging chamber. The gray dashed square indicates the field of view (103 μm × 103 μm); the dashed arrow represents the direction of aCSF flow through the chamber. The distance between the pipette tip and tanycyte bodies is 60 μm. 3 V, third ventricle; ARC, arcuate nucleus; DMH, dorsomedial hypothalamic nucleus; ME, median eminence; VMH, ventromedial hypothalamic nucleus. (**b, c**) Montage of the tanycyte Ca^2+^ response to direct puffs of 5 mM l-arginine and the traces from corresponding ROIs. Black bar on the ROI trace graph represents the duration of arginine puffing. Scale bar 20 μm. (**d**) Essential amino acids l-arginine an l-lysine elicited the highest responses at low concentrations. l-serine and l-alanine only exhibited strong responses when applied at a higher concentration (10 mM). l-proline, reported in literature as a weak agonist for Tas1r1/Tas1r3, triggered only a slight calcium wave at both concentrations. (**e**) ROI traces of the change in F_340_/F_380_ during typical responses to l-arginine, l-lysine and l-alanine. (**f**) No response to l-arginine was observed when the puff pipette was moved to apply l-arginine directly at the hypothalamic parenchyma, around 100 μm lateral to the apical surface of tanycytes (left). When the pipette was returned to puffing at directly at the tanycyte cell bodies, robust Ca^2+^ signals were evoked (right).

Amino acids and drugs were applied via either the bathing medium or series of 16 manual 300 ms puffs (pressure ∼0.75 bar) from a patch pipette with a 2–3 μm tip, applied roughly every second for Ca^2+^ imaging, or a single 700 ms puff for ATP biosensing. This series of short puffs, as established in prior work [17], superfuses the desired substance over the edge of the slice at a steady state concentration some 30- to 40-fold lower than the pipette concentration. The positioning of the brain slice and the pipette in the incubation chamber is shown in Figure 1a.

The amino acids used were l-alanine (Acros Organics, US), l-arginine (Acros Organics, US), l-lysine (Fisher Scientific, US), l-proline (Merck, Germany), and l-serine (Sigma–Aldrich, UK). The concentration of the amino acids in the puff pipette was 150–300 mM, depending on osmolarity, which was maintained around 300 mOsm to match aCSF. All amino acid solutions were made in pure H_2_O. 10 mM 4-(2-hydroxyethyl)-1-piperazineethanesulfonic acid (HEPES; Sigma–Aldrich, UK) buffer was added to 165 mM l-arginine. According to previous experiments using glucose biosensors placed at the edge of the brain slice during puffs of 300 mM glucose, the final concentration of glucose reaching the slice was 30–40 times lower than the concentration in the pipette depending on the distance from the tip of the pipette to the tanycytes [17]. As we were unable to obtain sensors for each amino acid, we used the glucose data to calculate the approximate concentrations of amino acids, and they will be referred to further in the text as: 5 mM and 10 mM l-alanine, 5 mM l-arginine, 5 mM l-lysine, 5 mM and 10 mM l-proline, 5 mM and 10 mM l-serine.

2 mM glycerol was added to the low glucose aCSF when using ATP biosensors.

The concentrations of the drugs used were as follows: 200 μM 10panx (Tocris Bioscience, UK), 200 μM 4-CPG (Tocris Bioscience, UK), 10 μM BBG (Sigma–Aldrich, UK), 10 μM and 100 μM CBX (Sigma–Aldrich, UK), 165 μM Gap26 (VWR International Ltd., UK), 0.5–1 mM IMP (Sigma–Aldrich, UK), 200 μM MAP4 (Tocris Bioscience, UK), 100 nM MRS2500 (Tocris Bioscience, UK), 50 μM Suramin (Sigma–Aldrich, UK), 30 μM PPADS (Tocris Bioscience, UK), 1 mM Probenecid (Sigma–Aldrich, UK), 50 μM Ruthenium Red (Sigma–Aldrich, UK). The solutions were prepared in aCSF except for Probenecid, which had to be dissolved in 1 mM NaOH and then neutralized with HCl prior to adding it to aCSF. Only one drug treatment was performed per slice to avoid loss of responses due to overstimulation, bleaching of Fura-2, or loss of cell viability.

Bath application of all substances except IMP, 10panx, and Gap26 included incubation in the flow chamber for 10–30 min without imaging. IMP was applied for 3–5 min to ensure it was available at the region of interest before the amino acid puffs. When using 10panx and Gap26, each slice was tested for a control response to 5 mM l-arginine, then taken out of the flow chamber and put into an incubation chamber with either regular low-glucose aCSF (control) or low-glucose aCSF with 10panx or Gap26 for 30 min, and put back into the flow chamber. Responses to l-arginine were then recorded within 5 min to prevent washout of the peptides.

### 2.3 Imaging

Prior to imaging, the slices were incubated for 75–95 min with 1.25 μM Fura-2 AM (Fura-2 dissolved in 20% Pluronic F-127 solution in DMSO, and diluted 1:800 into low-glucose aCSF; Life Technologies, UK). After incubation, the slices were transferred into a holding chamber with low-glucose aCSF to wash out excess Fura-2 AM and DMSO/pluronic. For imaging, they were transferred to a flow chamber on an Olympus BX51 microscope equipped with a 60× water immersion objective (NA 1.0). Ratiometric imaging of Fura-2 emissions at excitation wavelengths of 340 and 380 nm (provided by a Cairn Research Optoscan monochromator) was performed under the control of the MetaFluor software.

Brain samples for *Tas1r1* expression imaging were obtained from the German Institute of Human Nutrition Potsdam-Rehbruecke. We used the tissue of Tas1r1-Cre/eR26-tauGFP mice that expressed GFP at the site of Tas1r1 [22], [23]. The tissue was cut at 35 μm using Bright OTF 5000 cryostat and mounted on slides with VECTASHIELD (Vector Laboratories, US) containing DAPI. We used Leica SP5 confocal laser microscope for imaging and FIJI software for further analysis.

### 2.4 ATP biosensing

For direct biosensing of ATP in brain slices, we used custom made enzyme-coated 7 μm carbon fiber microelectrodes. A “null” electrode was inserted next to the biosensor and used as a reference for any mechanical or electrical disturbances during the recordings. The final values of the measurements were calculated by subtracting the “null” values from the biosensor values at each time point. The arrangement of the electrodes in the brain slice is shown in Figure 4a.

**Figure 2:**
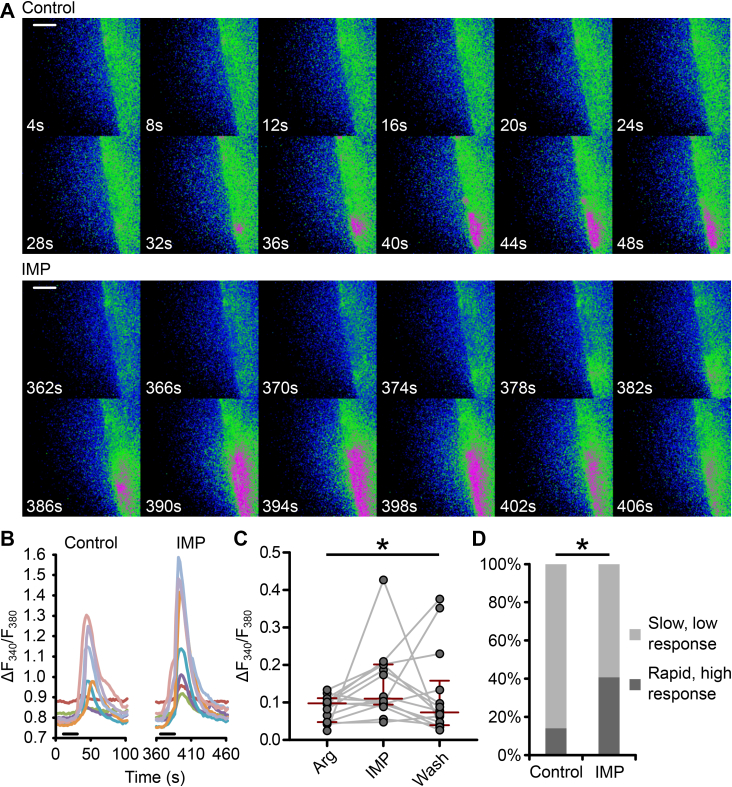
**IMP enhances tanycyte responses to****l****-arginine.** (**a, b**) Montage and graph of ROI traces demonstrate the change of the response of one slice to a series of 300 ms puffs of 5 mM L-arginine in normal aCSF (Control) and after the addition of 0.5–1 mM IMP to the bathing medium (IMP). Scale bars 20 μm. (**c**) IMP increased tanycyte responses to l-arginine (P = 0.016 Wilcoxon signed rank test, n = 14 slices (from 4 animals)). Black bars on the ROI traces represent the duration of amino acid puffing. (**d**) The proportion of cells exhibiting rapidly rising high responses (ΔF_340_/F_380_ > 0.15) increases from 14.1% to 40.9% with added IMP (P = 0.038, G test, n = 15 slices (from 4 animals)).

**Figure 3:**
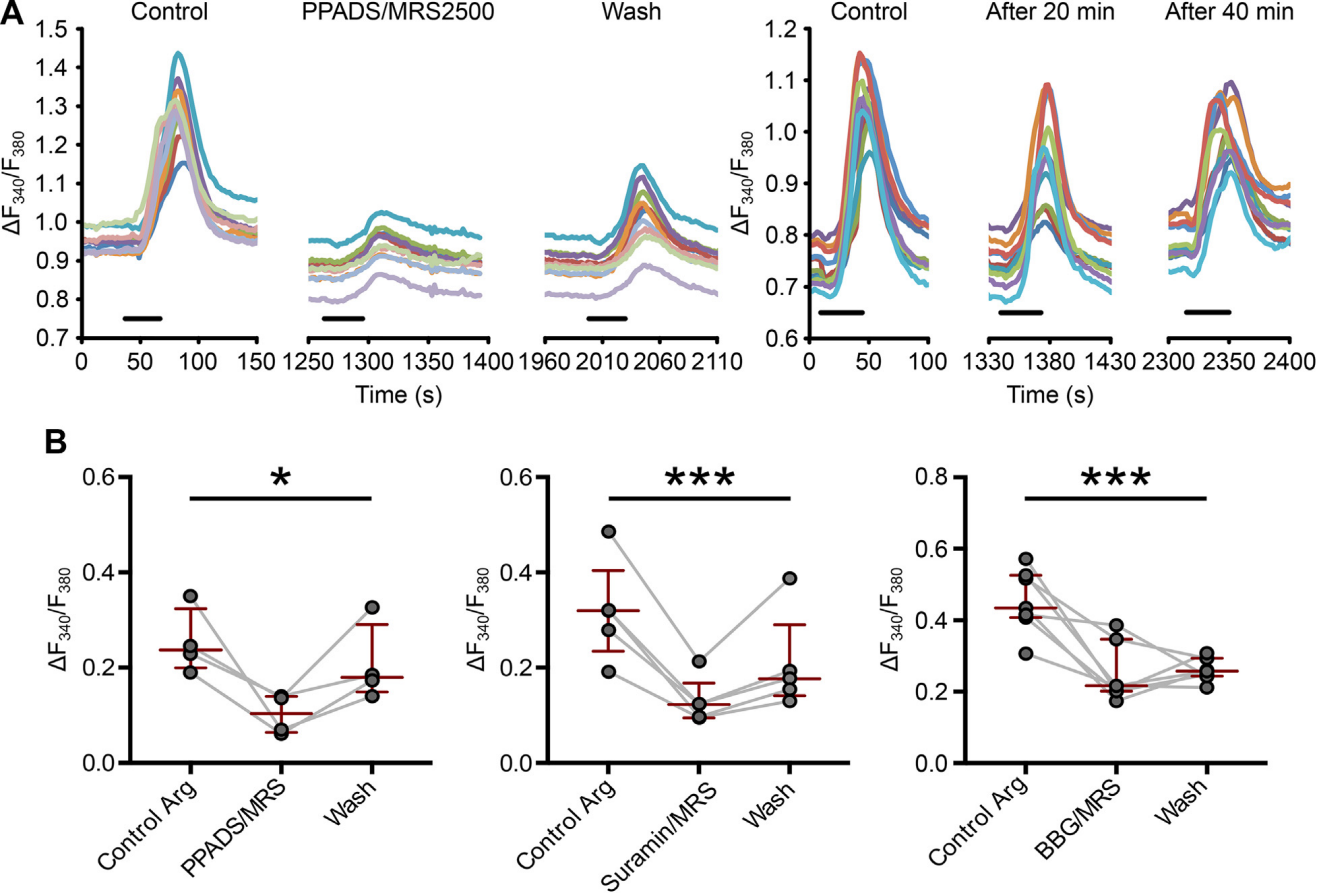
**P2X and P2Y**_**1**_**receptors take part in the tanycyte amino acid detection pathway.** (**a**) Comparison of ROI traces: the effect of a combination of PPADS and MRS2500 on tanycyte responses to l-arginine vs. time-matched control. (**b**) P2X and P2Y receptor antagonist combinations reduced tanycyte responses to l-arginine. PPADS/MRS2500 block P2Y_1_ and most P2X receptors except P2X_4_ and P2X_6_ (P = 0.0417, Friedman test, n = 4 slices (from 2 animals)). Suramin/MRS2500 block the same receptors as PPADS/MRS2500 with more specificity for P2X_2_ (P = 0.0008, Friedman test, n = 5 slices from 2 animals). BBG/MRS2500 block P2Y_1_, P2X_2_ and P2X_7_ (P = 0.0027, Friedman test, n = 7 slices (from 4 animals)).

**Figure 4:**
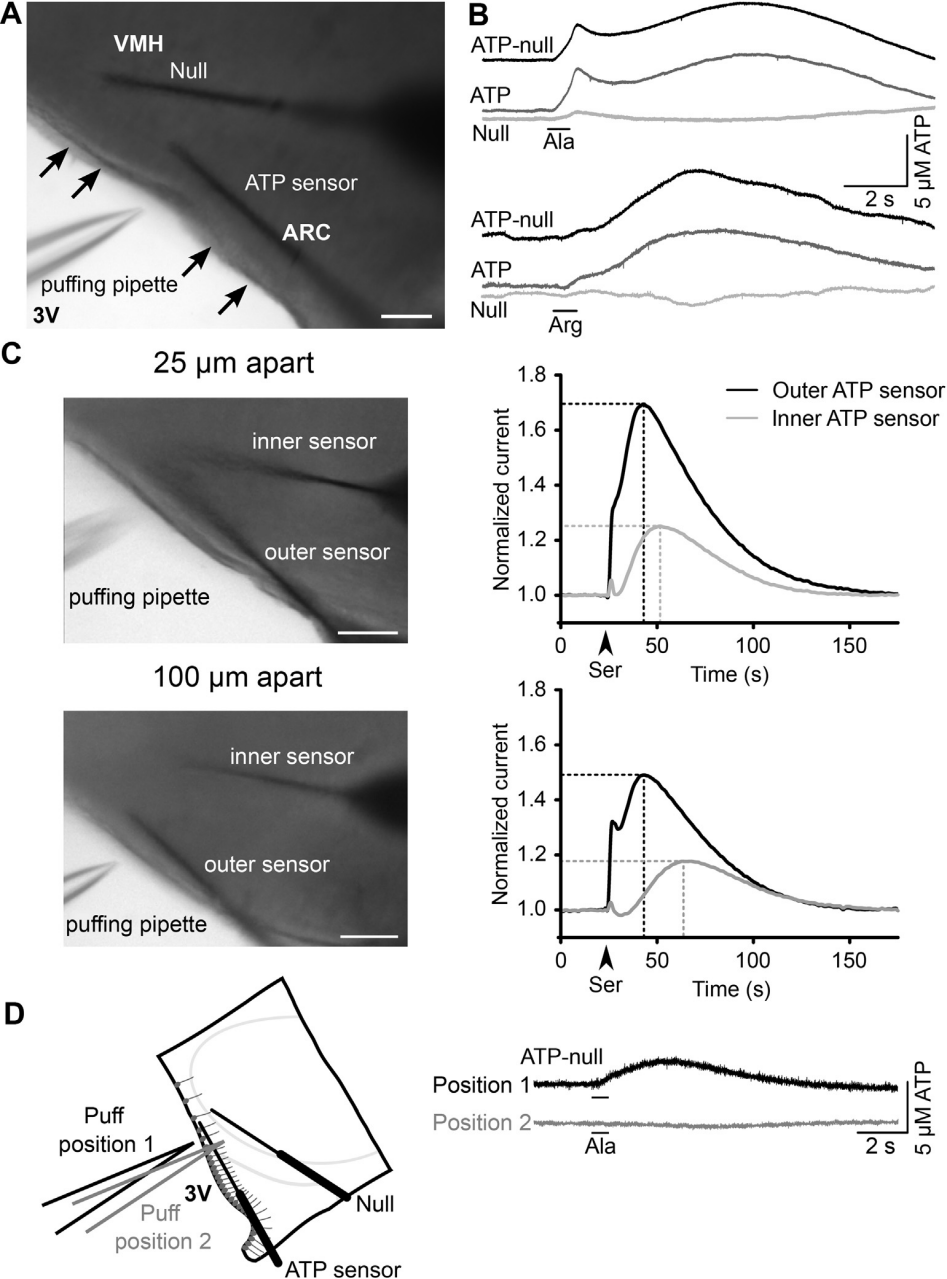
**Tanycytes release ATP in response to amino acids.** (**a**) The biosensor setup. The arrows indicate the tanycyte layer. 3 V, third ventricle; ARC, arcuate nucleus; VMH, ventromedial hypothalamic nucleus. (**b**) Example ATP biosensor traces from tanycytes responding to l-alanine and l-arginine. (**c**) ATP travels down the tanycyte processes and into the brain parenchyma. The graphs show the biosensor current normalized to the background current for responses to l-serine between two biosensors placed 25 and 100 μm apart. Scale bars 100 μm. (**d**) Example ATP biosensor traces from tanycytes when l-alanine is applied on tanycytes or directly on hypothalamic parenchyma with a diagram indicating the biosensor and puff pipette arrangement. The same results were obtained in 3 different slices.

The biosensor and the “null” were connected to Sycopel Duo-Stat ME 200+ potentiostat. The biosensor traces were recorded via a Data Translation DT3016 AD board and custom software was used for storage and analysis.

2 mM glycerol was added to all the bathing solutions used in these experiments to allow the enzymes within the biosensor – glycerol kinase and glycerol-3-phosphate oxidase – produce hydrogen peroxide when ATP is present (Supplementary Figure 1) [24].

The biosensors were calibrated with 50 μM ATP solution in aCSF at the start and the end of each day, as well as in the middle of the experiments.

### 2.5 Data analysis

The emission ratios for F_340_/F_380_ were calculated using ImageJ software for every individual tanycyte visible in the field. The baseline was calculated from 15 images prior to the start of the amino acid application and maximum change in the intensity in each region of interest was found for every trial (when substances potentially altering tanycyte responses were used, trials were described as follows: CONTROL amino acid application; DRUG; and WASH, recovery after washing the slice in regular aCSF). Average responses were then calculated for each slice.

The data from the biosensor recordings were analyzed using proprietary software. The amplitudes of the response peaks were measured manually and then compared to the amplitude of the biosensor calibration curve recorded using a known amount of ATP (50 μM) to calculate the amount of ATP detected for each response.

Graphpad Prism 7 was used to produce graphs. All data represented in the graphs can be found in the Supplementary Data File.

### 2.6 Statistical analysis

Statistical analysis was performed using Graphpad Prism 7 software. For experiments looking at effects of drugs, a single brain slice was considered as an independent replicate (the total number of animals that the slices were taken from is given in brackets). The Friedman non-parametric 2 way ANOVA was used to compare the responses across the CONTROL, DRUG, and WASH trials. Median responses in both Ca^2+^ imaging and ATP biosensing experiments were compared between the CONTROL and the DRUG trial using Wilcoxon's matched paired signed ranks test. The WASH trial was used to evaluate the loss of cell vitality and responsiveness due to time and the number of amino acid applications. Exact probabilities of the tests are reported in the text. The G test was used to evaluate the significance of the change in the proportion of high responses (ΔF_340_/F_380_ > 0.15) in the IMP experiment.

In the *Tas1r1*-null versus wild type comparisons, a single animal was considered to be a replicate. On average recordings were made from 2 brain slices per animal for each amino acid, and the results averaged to give a single value per animal. The Mann–Whitney *U* test was used to compare the responses between wild type and *Tas1r1*-null animals. For all experiments involving *Tas1r1*-null animals, a power calculation was performed after gathering the initial data to determine the appropriate sample sizes.

All statistical tests were two-sided. Data collection and analysis were not performed blind to the conditions of the experiments. Randomization was not performed as mouse litter sizes were small, and all littermates were used.

## 3 Results

### 3.1 Tanycytes respond to amino acids

To examine whether tanycytes can respond to amino acids, we used Ca^2+^ fluorescence imaging to assess responses to two essential amino acids, l-arginine (Arg) and l-lysine (Lys), and three non-essential amino acids, l-alanine (Ala), l-serine (Ser), and, l-proline (Pro). As shown in Figure 1(b–e), tanycytes responded to 30 s periods of direct amino acid puffs from a patch pipette (Figure 1a) with a wave of Ca^2+^. Typical responses to Lys, Arg and Ala are shown in Supplementary movies. Both essential amino acids evoked greater responses at lower concentrations compared to the non-essential amino acids. Pro did not elicit responses (Figure 1d). Tanycytes only responded to amino acids when they were directly applied to the apical surface of the cells: puffing Arg onto the hypothalamic parenchyma (100 μm lateral to the edge of the 3rd ventricle) did not elicit Ca^2+^ release (example trace in Figure 1f; median ΔF_340_/F_380_ 0.014, interquartile range (IQR) 0.006–0.067 n = 5 slices (from 4 animals)). This selective responsiveness of tanycytes to stimulation of their ventricle-facing apical surface is similar to that which we have previously reported for their sensitivity to glucose [17].

The following are the Supplementary data related to this article:

Supplementary Movie 1 Tanycyte response to lysine puff. Supplementary Movie 1

Supplementary Movie 2 Tanycyte response to arginine puff. Supplementary Movie 2

Supplementary Movie 3 Tanycyte response to alanine puff. Supplementary Movie 3

The sensitivity of tanycytes to different amino acids is relatively consistent with involvement of the Tas1r1/Tas1r3 umami receptor originally described in the taste buds of the tongue [20], [21]. A further distinctive property of Tas1r1/Tas1r3 is the ability of the allosteric modulator inosine 5′-monophosphate (IMP) to enhance responses to amino acids. IMP is a compound of umami taste known to enhance the umami taste properties in a concentration-dependent manner (for 0.1–10 mM) by stabilizing the closed confirmation of the Tas1r1 subunit after binding of an amino acid [25]. Introducing IMP (0.5–1 mM) into the bathing media prior to Arg puffs resulted in higher amplitude of tanycyte responses (Figure 2a–c), as well as an increase in the number of cells exhibiting rapid, high responses (threshold of ΔF_340_/F_380_ > 0.15 based on observation; Figure 2d).

The amino acid sensitivity profile and the potentiation of the Ca^2+^ signal with IMP revealed that tanycyte responses to amino acids shared some characteristics with those expected of Tas1r1/Tas1r3.

### 3.2 Amino acid responses are mediated via ATP receptors

The responses to amino acids in taste receptor cells of the tongue follow an ATP receptor-dependent pathway [26]. Functional evidence demonstrates that P2Y_1_ receptors are present in tanycytes and can elicit Ca^2+^ signals [17], [19]. Thus, we first tested whether the potent and selective P2Y_1_ receptor antagonist MRS2500 could alter tanycyte responses to amino acids. We found that MRS2500 by itself had no effect on the Ca^2+^ responses evoked by Arg (Supplementary Figure 2a and b). This contrasts with its ability to substantially block the responses to glucose and sweet tasting compounds [19]. We tried a further range of antagonists: PPADS, Suramin, and brilliant blue G (BBG; Supplementary Figure 2c), which have actions at several P2X and P2Y receptors. Individually, none of these antagonists were effective in reducing the Ca^2+^ signal evoked by Arg. However, a combination of any P2X antagonist and MRS2500 successfully reduced tanycyte responses to Arg (Figure 3). This suggests that multiple ATP receptors were required to produce a Ca^2+^ wave in response to Arg, with P2Y_1_ definitely being one of them. The lack of effect from individual antagonists, therefore, may be explained by the multiple receptors involved in mediating the response and the fact that blocking only one at a time is unable to significantly reduce the response to Arg.

To directly study amino acid-evoked ATP release from tanycytes, we used ATP microelectrode biosensors (see Supplementary Figure 1 for the mechanism of ATP biosensing). We inserted the biosensors into the hypothalamic parenchyma close to the tanycyte somata (Figure 4a). We found that Ala, Arg, and Ser were all able to evoke robust ATP release (Figure 4b, c). We tested whether tanycytes could release ATP from their processes by placing a second ATP biosensor at a varying distance from tanycyte cell bodies. This second ATP biosensor recorded ATP release that was delayed with respect to the biosensor closer to the tanycyte cell bodies. The wave of ATP traveled into the parenchyma at an average speed of 3.6 μm/s (SD = 1.48, n = 5), which is similar to the speed at which Ca^2+^ travels down the tanycyte processes [19] (Figure 4c).

To test whether the release of ATP depended on stimulation of the tanycyte cell bodies, we applied Ala onto hypothalamic parenchyma, with the biosensors placed close to the tanycyte cell bodies. With this arrangement, we saw no release of ATP demonstrating the requirement for specific activation by amino acids of the tanycyte cell bodies (Figure 4d). This is consistent with the lack of Ca^2+^ signals evoked by parenchymal puffing (Figure 1f), supporting the hypothesis that activation of tanycytes is necessary for the observed ATP release. We cannot exclude that downstream activation of astrocytes, for example, could secondarily contribute to the ATP release recorded in the parenchyma.

### 3.3 The ATP release is channel-mediated

We next examined the mechanism of amino acid-evoked ATP release from tanycytes. In taste receptor cells of the tongue, a number of different channels have been suggested as being the conduit for ATP release including pannexin 1 channels (Panx1) [27] and calcium homeostasis modulator 1 (CalHM1) [28]. ATP release via connexin 43 (Cx43) has been proposed to be crucial for tanycyte glucosensing [18].

**Figure 5:**
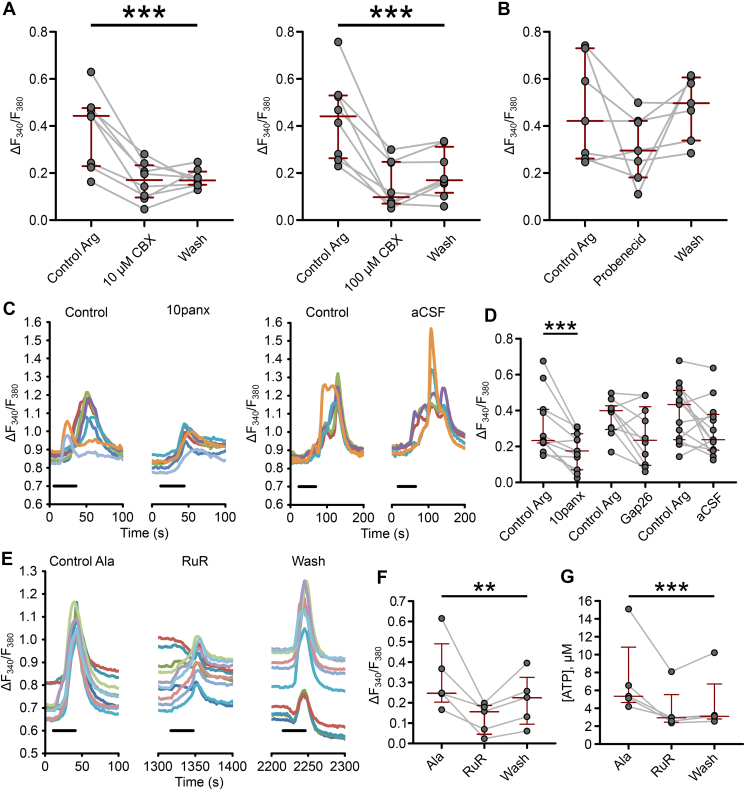
**Tanycyte ATP release is channel-mediated.** (**a**) Non-specific hemichannel antagonist carbenoxolone (CBX) reduced tanycyte responses to l-arginine at 10 and 100 μM (10 μM, P = 0.0009, Friedman test n = 9 slices (from 3 animals); 100 μM CBX, P = 0.0003, Friedman test, n = 8 slices (from 4 animals)) (**b**) Probenecid, a pannexin antagonist, did not have an effect (P = 0.1147, Friedman test, n = 7 slices (from 5 animals)). (**c, d**) ATP is most likely released via Pannexin 1 after l-arginine application. The pannexin 1 mimetic peptide 10panx weakened the responses (P = 0.0029, Wilcoxon signed ranks test, n = 11 slices (from 4 animals)). Gap26, the mimetic peptide for connexin 43, showed no such reduction. (**e–g**) Ruthenium red, which blocks CALHM1, reduced tanycyte responses to l-alanine. Tanycyte responses to l-alanine lead to ATP release trough CALHM1(P = 0.0085, Friedman test, n = 5 slices (from 2 animals)). The reduction in the response was also observed with direct ATP biosensing (P = 0.0008, Friedman test, n = 5 slices (from 3 animals)).

We found that both high (100 μM) and low (10 μM) concentrations of carbenoxolone (CBX) reduced tanycyte responses to Arg (Figure 5a). The lower dose of CBX has some selectivity for Panx1 compared to Cx43 [27]. We next tested probenecid, reported to have selectivity for Panx1; however, this had little effect on the responses to Arg (Figure 5b). We then employed mimetic peptides, which have greater selectivity: 10panx to block Panx1 and Gap26 to block Cx43. 10panx greatly reduced the response to Arg, however Gap26 have no effect (Figure 5c, d). Therefore, we conclude that Panx1 but not Cx43 contributes to the ATP release evoked by Arg.

To test the possible involvement of CalHM1 as an additional mechanism for tanycyte ATP release we used Ruthenium Red (RuR; Figure 5e). While RuR did not affect the responses to Arg (Supplementary Figure 3), testing it against Ala revealed a strong reduction of the Ca^2+^ signal (Figure 5e, f), suggesting that the two channels, Panx1 and CalHM1, could release ATP in response to different amino acids. We repeated this test using ATP biosensors to measure ATP release directly. We found that RuR reduced tanycyte ATP release evoked by Ala (Figure 5g). This strengthens the evidence that activation of tanycytes is essential for the amino acid-evoked ATP release.

### 3.4 Multiple umami taste receptors are involved in tanycyte amino acid detection

**Figure 6:**
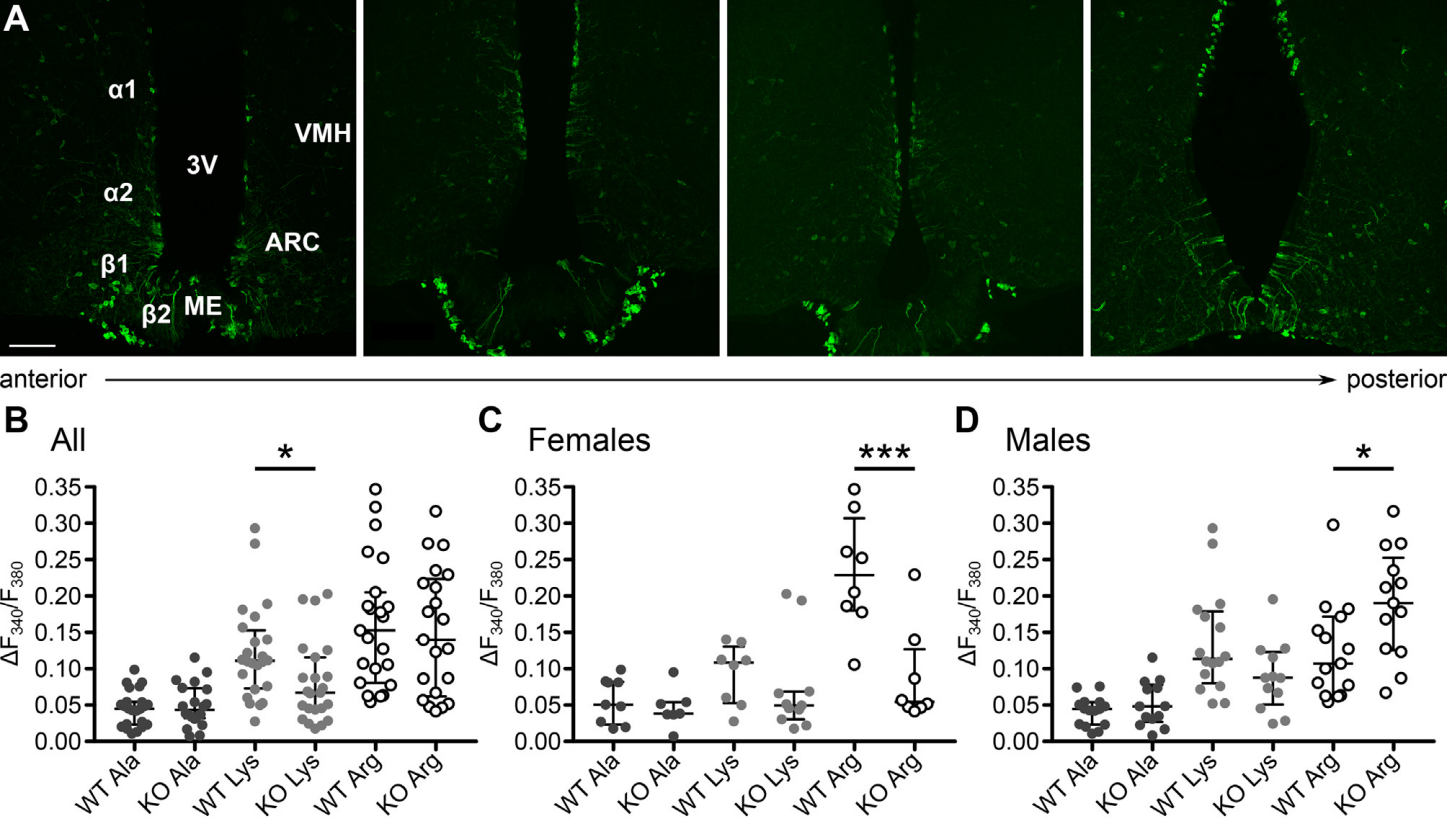
**Tas1r1/Tas1r3 is involved in tanycyte amino acid sensing.** (**a**) The hypothalamic tissue from Tas1r1-Cre/eRosa26-tauGFP mice shows that the Tas1r1 subunit is expressed in some, but not all hypothalamic tanycytes, as well as some neurons and ependymal cells. The expression of Tas1r1 is consistent in both anterior (images on the left) and posterior (images on the right) regions of the third ventricle. Scale bar 100 μm. (**b**) *Tas1r1*-null mice showed no change in the responses to l-alanine or l-arginine but a reduction in the responses to l-lysine (P = 0.0147, Mann–Whitney *U* test, n = 23 animals). (**c**) Female mice lacking Tas1r1 exhibit strongly reduced responses to l-arginine (P = 0.003, Mann–Whitney *U* test, n = 8 animals). (**d**) In male *Tas1r1*-null mice, tanycytes are more sensitive to l-arginine (P = 0.017, Mann–Whitney *U* test, n = 15 animals).

In taste buds, multiple receptors are involved in detecting amino acids. Therefore, we sought to directly test the role of Tas1r1/Tas1r3 by using a binary genetic strategy [22], [23]. Tas1r1-Cre mice were bred with Rosa26-tauGFP reporter mice. In the offspring, the tauGFP reporter labels cells normally expressing the *Tas1r1* gene. Confocal imaging of the hypothalami of these mice showed GFP expression in some but not all tanycytes, a few neurons, and some ependymal cells, especially in the posterior regions (Figure 6a).

Therefore, we examined Ca^2+^ signaling in tanycytes of *Tas1r1*-null mice to test whether their responses to amino acids were altered. These mice had a reporter construct, consisting of a truncated barley lectin followed by an internal ribosome entry site and mCherry, knocked into the Tas1r1 locus [29].

We found that the *Tas1r1*-null mice (males and females analyzed together) showed no difference in the responses to Ala (Figure 6b). There was a striking variation in the responses to Lys and Arg. The responses to Lys were partially reduced (P = 0.0147 Mann–Whitney *U* test), but there was no change in the responses to Arg (Figure 6b).

To better understand the variability of the phenotype in the *Tas1r1*-null mice, we analyzed the effects of age on tanycyte sensitivity to amino acids, but found no relationship between age and amplitude of the responses to any of the tested amino acids within our range (Supplementary Figure 4). We then separated the responses by sex (Figure 6c). In neither males nor females were the responses to Ala altered by deletion of the *Tas1r1* gene. The responses to Arg were strongly reduced in female *Tas1r1*-null mice. However, in male mice, the responses to Arg were enhanced in the knock out compared to the wild type. This suggests that males can compensate for the loss of the *Tas1r1* gene (possibly via upregulation of another receptor), whereas female mice cannot. Interestingly in wild type mice, tanycytes in females were more sensitive to Arg than those in males (P = 0.0025, Mann–Whitney *U* test). This correlates with the differing effect of loss of the *Tas1r1* gene in the responses to Arg in tanycytes from male and female mice. While it has not to our knowledge been reported before, the biological significance of this difference between the sexes remains unclear.

**Figure 7:**
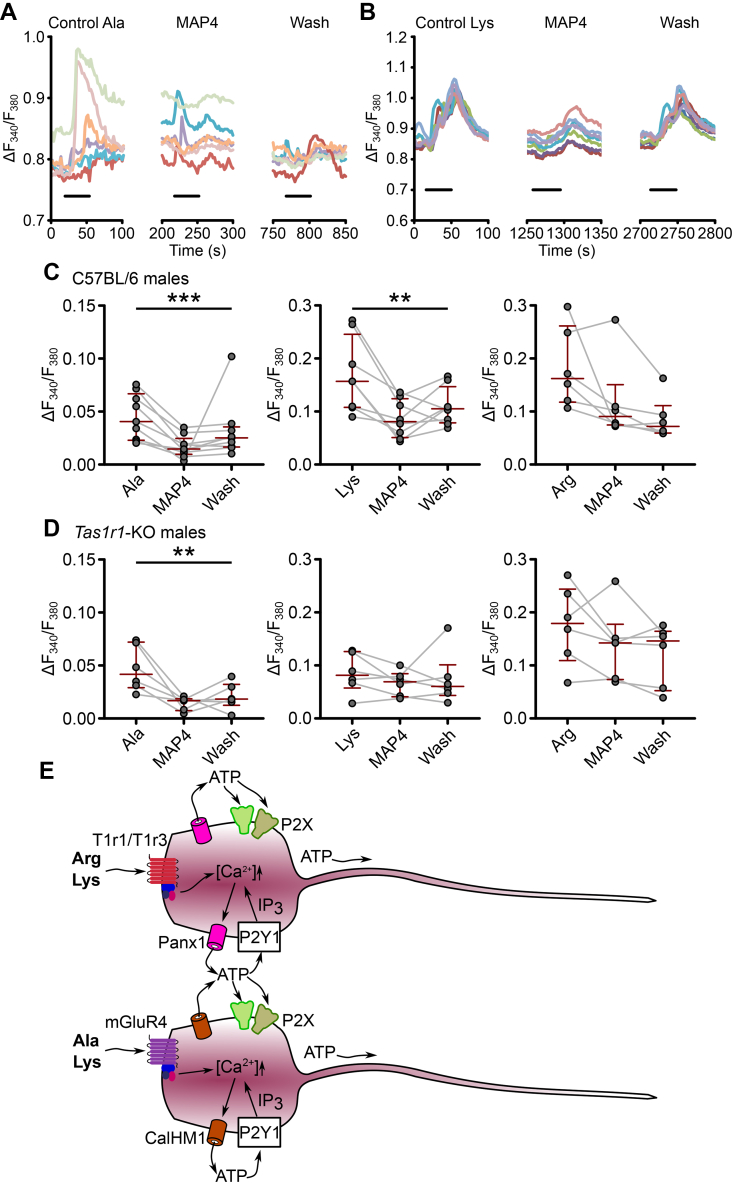
**Tanycytes use mGluR4 in addition to Tas1r1/Tas1r3 to detect****l****-amino acids.** (**a, b**) Example ROI traces of the effects of the specific mGluR4 antagonist MAP4 on l-alanine and l-lysine. (**c**) MAP4 blocked tanycyte responses to l-alanine (P = 0.0007, Friedman test, n = 9 slices (from 6 animals)); the responses to l-lysine were also reduced (P = 0.0099, Friedman test, n = 8 slices (from 7 animals)). l-arginine responses were unaffected. (**d**) Using MAP4 on the *Tas1r1*-null mice did not eliminate all tanycyte responses to l-amino acids. The effect on l-alanine remained (P = 0.0055, Friedman test, n = 6 slices (from 6 animals)), while any effect on l-lysine was masked due to lower initial response (P = 0.7402, Friedman test, n = 6 slices (from 6 animals)). There was still no change in the responses to l-arginine. (**e**) The proposed pathway for tanycyte amino acid sensing.

Since Ala responses were unaffected by the deletion of *Tas1r1*, we investigated whether other known umami taste receptors, mGluR1 and mGluR4 [30], might be involved. Whereas the mGluR1 antagonist S-4-carboxyphenylglycine (4-CPG) [31] did not have any effect on Ala responses, S-2-amino-2-methyl-4-phosphonobutanoic acid (MAP4), a specific mGluR4 antagonist [32], greatly reduced tanycyte responses to Ala and partially reduced the responses to Lys (Figure 7a–c). MAP4 had no effect on the responses to Arg. To see whether a combination of MAP4 and *Tas1r1* gene deletion had a more complete effect on amino acid responses, we looked at the effect of MAP4 in male *Tas1r1*-null mice. The effect on tanycyte Ala sensitivity remained strong (Figure 7d), but no effect of MAP4 was observed on responses to either Lys or Arg. Any potential effect of MAP4 on responses to Lys may have been masked by the control Lys responses being lower in the *Tas1r1*-null mice (P = 0.02, Mann–Whitney *U* test). Nevertheless, if mGluR4 had been the only receptor mediating the responses to Lys in mice lacking the Tas1r1/Tas1r3 receptor, we would have expected a more complete reduction of the responses under MAP4. It seems unlikely therefore that male mice compensate for the loss of the *Tas1r1* gene by upregulating mGluR4.

Our results indicate at least two mechanisms of tanycyte amino acid sensing via umami taste receptors: Tas1r1/Tas1r3 for Arg and Lys, and mGluR4 for Ala and Lys (Figure 7e). Interestingly, the Allen Mouse Brain Atlas shows expression of mGluR4, by *in situ* hybridization, in the ependymal layer surrounding the 3rd ventricle in the region of the median eminence [33].

## 4 Discussion

### 4.1 Tanycyte umami taste signaling

Tanycytes detect amino acids, and they do so through at least two receptors, Tas1r1/Tas1r3 and mGluR4. While tanycyte glucosensing was long suspected, tanycyte amino acid sensitivity has not been foreshadowed in literature. The direct detection of amino acid concentration changes in the third ventricle by tanycytes, therefore, is the first known non-neuronal mechanism of amino acid sensing in the brain.

Our work shows that tanycytes functionally express Tas1r1/Tas1r3, and the loss of the Tas1r1 subunit of the receptor in mice affects tanycyte responses to some amino acids, depending on the sex of the mouse. Given that Tas1r1 expression is sparse (Figure 6a), the partial effects of knocking out this subunit are perhaps not surprising. The sex differences in the adaptation to the loss of Tas1r1 are consistent with the emerging literature on energy homeostasis in male and female mice. Differences have been observed in POMC production in response to a high-fat diet [34] and the effects of changes in diet on hypothalamic neurogenesis [35], [36].

Furthermore, in our experiments IMP enhanced tanycyte responses to arginine, providing further support for the involvement of Tas1r1/Tas1r3 in tanycyte amino acid sensitivity.

As the mGluR4 antagonist MAP4 greatly reduced responses to Ala and partially blocked those to Lys, mouse tanycytes also use mGluR4 to detect these two amino acids. This multiplicity of receptors used by tanycytes to detect amino acids suggests that this is a physiologically important mechanism and reflects the current knowledge in the field of umami taste research. There has been an on-going discussion between various groups investigating human and rodent taste receptors about the nature and identity of the umami taste receptor; while a significant body of evidence pointed towards Tas1r1/Tas1r3, knocking out the Tas1r3 subunit of this receptor in mice did not eliminate all responses to umami-tasting amino acids [30], [37]. The current consensus seems to be that additional receptors such as mGluR1 and mGluR4 can sense a range of amino acids in the taste receptor cells in the tongue, and these mechanisms can compensate for each other when one of the receptors is lost [38].

It is also possible that due to the similarities between Tas1r1/Tas1r3 and Tas1r2/Tas1r3, the sweet taste receptor could adapt to respond to some amino acids in rodents lacking the *Tas1r1* gene, which would explain why we still saw some responses to arginine and lysine in the *Tas1r1*-null mice when mGluR4 was blocked (see Figure 7d).

### 4.2 Similarities between tanycytes and taste receptor cells

The parallel between tanycyte and taste receptor cell amino acid sensing extends beyond the use of umami taste receptors. Firstly, in type II taste receptor cells in the tongue, ATP is used as a neurotransmitter to send a signal about the taste qualities of a substance to sensory nerve fibers, which use P2X_2_ and P2X_3_ receptors to detect it [26]. ATP also exerts positive autocrine feedback onto receptor cells by activating P2Y receptors to enhance ATP release [39]. We have provided sound evidence that tanycytes respond to amino acids with increased Ca^2+^ and the release of ATP, and the spread of the ATP signal requires P2X and P2Y receptors. By measuring ATP release from tanycytes directly using microelectrode biosensors, we have shown not only that tanycytes respond to amino acids by producing a wave of ATP but also that a wave of ATP passes into the brain parenchyma. Hypothalamic neurons are known to express both P2X and P2Y receptors that could sense the tanycyte signals [40], [41], [42]. This reveals potential for tanycyte-to-neuron signaling, allowing tanycytes to inform the arcuate nucleus and the rest of the hypothalamus about amino acid availability in the third ventricle.

The need for multiple P2 receptor antagonists to stop the spread of the ATP signal was unexpected. Experiments on tanycyte sweet taste sensing previously showed that inactivating P2Y_1_ with MRS2500 was sufficient to reduce tanycyte responses to glucose and artificial sweeteners mediated by Tas1r2/Tas1r3 [19]. This was not the case for tanycyte amino acid detection. Our mix of antagonists blocked a number of P2X receptors including P2X_2_ and P2X_3_, as well as P2Y_1_, so while ATP signaling in tanycytes is not exactly the same as in the tongue, the involvement of a higher number of receptors for ATP could be an adaptation to increase sensitivity as the relative levels of amino acids in the third ventricle are much lower than they are in the oral cavity during a meal.

Secondly, tanycyte responses are mediated by Panx1 and CalHM1, both of which have been reported to be involved in umami taste signaling in mice [27], [28]. The evidence for the two channels in taste receptor cells has been contradictory, as different groups have shown strong effects of blocking or knocking out either channel on taste transduction [28], [43], [44]. We demonstrate in our study that tanycytes use both Panx1 and CalHM1, and they seem to be downstream of different umami taste receptors; activation of Tas1r1/Tas1r3 with arginine leads to opening of Panx1, while activation of mGluR4 with alanine induces ATP release through CalHM1.

The detection of circulating nutrients by tanycytes, thus, is remarkably similar to that of taste sensing in the tongue both in the types of receptors involved and the consequent downstream signaling via channel-mediated release of ATP. Thus, tanycytes should be thought of as general nutrient sensors in the hypothalamus.

### 4.3 Amino acid detection in the brain

Amino acids from ingested food enter the bloodstream and can cross the blood–brain barrier in the region of the median eminence, which has increased permeability, to enter the hypothalamus [45]. There are several different transporter systems that take the blood plasma amino acids across the blood–brain barrier: System L for neutral amino acids with large side chains; System y^+^ for sodium-independent uptake of cationic amino acids; System x^−^ for anionic amino acids; System N for sodium-dependent uptake of l-glutamine, l-histidine and l-asparagine; Systems A and ASC for small neutral amino acids; System B^o+^ for neutral and basic amino acids; and System X^−^ for l-glutamate and l-aspartate [46], [47].

After a meal, the concentrations of dietary amino acids increase in both blood plasma [48] and CSF [49]. The baseline levels of amino acids in rat CSF vary from 1 to 5 μM for isoleucine, proline, tryptophan, cysteine, and aspartate to up to 500 μM for glycine [50]. Experiments in freely moving rats have shown an increase of alanine, isoleucine, leucine, methionine, threonine, tyrosine, and valine concentrations in the lateral hypothalamic area within 20–40 min of dietary amino acid gavage [51]. CSF concentrations of all these amino acids plus asparagine and glycine in the medial preoptic area reflect dietary protein content [52]. The concentrations of the two essential amino acids used in our study, arginine and lysine, only increase by a small amount after feeding [51], but the combination of multiple amino acids ingested at once and the potentiation of the Tas1r1/Tas1r3 receptor by circulating nucleoside monophosphates such as IMP could be sufficient to evoke tanycyte signaling.

There are several neuronal mechanisms of direct amino acid sensing that have already been described in the brain. Some neurons (around 90% of NPY/AgRP and 45% of POMC) in the mediobasal hypothalamus express mTOR, which is indirectly activated by amino acids and has been described as an ATP sensor [2], [53]. ICV administration of l-leucine, a potent activator of mTOR, reduces the levels of NPY in ARC, produces anorexia and causes weight loss, while l-valine, similar in structure but unable to activate mTOR, does not have such an effect [2]. It is important to note that Tas1r1/Tas1r3 has been shown to play a part in the mTOR pathway [54]. mTOR signaling is also involved in amino acid sensing in the brainstem nucleus of the solitary tract, which integrates signals from the gastrointestinal tract and has an influence on food intake [55]. Hao et al. have found evidence that the accumulation of uncharged tRNA is the main signal of essential amino acid deficiency in the anterior piriform cortex [56]. Hypocretin/orexin neurons of the lateral hypothalamus are sensitive to non-essential amino acids and respond to a dietary mix of amino acids using a mechanism that involves K_ATP_ and System A amino acid transporters [5].

Our discovery that tanycytes also sense amino acids in the CSF via at least two receptors is an important advance that suggests: 1) that tanycytes are anorexigenic; and 2) that they might act with the neural networks in the hypothalamus to regulate food intake. This could potentially be via the wave of ATP release that travels down the tanycyte processes into ARC, as ARC neurons express P2 receptors [57], [58]. The ATP from tanycytes could potentially excite the anorexigenic POMC neurons in the ARC or activate NPY/AgRP neurons via mTOR (tanycytes have been shown to have a close anatomical association with NPY neurons [59]) to inhibit NPY secretion and suppress appetite this way.

### 4.4 Potential role for amino acid sensing in tanycyte neurogenesis

Hypothalamic tanycytes form one of the three neurogenic niches described so far in the adult brain [60]. Several studies have shown that neurogenesis in the hypothalamus is responsive to changes in diet [61], [62]. It is also known that the newly produced neurons migrate into the arcuate nucleus and are incorporated in the POMC and AgRP circuits [63].

P2Y receptor signaling is required to induce neural stem cell proliferation [64]. Our results show that P2Y_1_ is one of the receptors in tanycytes that is activated by ATP release following amino acid or glucose application. It is therefore tempting to speculate that sustained activation of tanycytes over a long period via amino acids or glucose could lead to a higher probability of proliferation.

Male mice fed a high-fat diet for an extended period of time show an increase in the generation of POMC neurons in the hypothalamus, most likely acting through G-protein-coupled receptor 40 (GPCR40) [65]. Research in female mice demonstrates that both high-fat and low-protein diets can reduce neurogenesis in the arcuate nucleus but increase the number of new cells in the median eminence [35]. The mechanism by which a low-protein diet could affect tanycyte proliferation in female mice has not been defined yet. However, we speculate that tanycyte amino acid sensing via umami taste receptors could help to maintain normal levels of neurogenesis in the hypothalamus, and that changes in protein content in the diet would alter the number of newly generated neurons.

Together, direct tanycyte-to-neuron signaling about amino acid availability and amino acid/ATP-induced tanycyte proliferation into arcuate neurons could represent a short-term and a long-term role for tanycytes in metabolic control. Our work provides a new piece of knowledge in the complicated subject of hypothalamic regulation of energy homeostasis. A more detailed understanding of how food intake and energy expenditure are determined in the brain may lead to the development of new strategies for overcoming the obesity epidemic and other metabolic disorders.

## 5 Conclusions

Hypothalamic tanycytes are directly sensitive to a range of essential and non-essential amino acids, which are important signals of satiety. Amino acids act on tanycytes via two receptors and trigger mobilization of intracellular Ca^2+^ followed by the release of ATP, which activates P2 receptors to further amplify the Ca^2+^ signal. The amino acid evoked ATP release travels into the parenchyma at sufficient concentrations to be capable of activating P2 receptors on hypothalamic arcuate neurons. Our data warrant investigation as to whether tanycytes may be physiological mediators of satiety signals and act to reduce food intake.

## Appendix A Supplementary data

Supplementary Table 1 The design and experimental logic of the study. Supplementary Table 1

Supplementary Figure 1 **The mechanism of ATP detection with microelectrode biosensors.** (**a**) Enzymatic scheme of the ATP biosensor. The cascade of enzyme reactions produces H_2_O_2_ when ATP is present. Glycerol is added to the bathing medium prior to ATP measurements. H_2_O_2_ is oxidized on the electrode surface (polarized to 500 mV). (**b**) The Null sensor is an exact copy of the ATP biosensor without the enzymes required for ATP detection. It is therefore only sensitive to electrical disturbances and non-specific events, allowing exclusion of any noise or artefacts from the ATP biosensor recording [24]. Supplementary Figure 1

Supplementary Figure 2 **l****-arginine-evoked Ca**^**2+**^**signals in tanycytes are not blocked by application of single P2X or P2Y receptor antagonists.** (**a**) Example ROI records of responses to arginine with and without 100 nM MRS2500. (**b**) Summary data for effect of MRS2500 on Arginine responses. (**c**) Summary data for effect of PPADS (30 μM), suramin (50 μM) or Brilliant Blue G (BBG, 10 μM) on arginine responses. Supplementary Figure 2

Supplementary Figure 3 **Tanycyte responses to****l****-arginine are not mediated by CALHM1.** The responses to l-arginine were unaffected by 50 μM Ruthenium Red. Supplementary Figure 3

Supplementary Figure 4 **Mouse tanycyte responses to****l****-amino acids do not change between the age of 5 and 16 weeks.** In both wild type and *Tas1r1*-null mice used in our experiments, there was no correlation between the age of the animals and the amplitude of their responses to l-alanine, l-lysine or l-arginine. Supplementary Figure 4
