# Supplementary material for: Amino acid sensing in hypothalamic tanycytes via umami taste receptors
Source: Mol Metab. 2017 Sep 14;6(11):1480–92. doi: 10.1016/j.molmet.2017.08.015 (PMC5681271; doi:10.1016/j.molmet.2017.08.015)
Supplement: Supplementary Table 1 — The design and experimental logic of the study. [file mmc1.pdf]

Question	Approach	Results	Controls	Figures
A. Do tanycytes respond to amino acids?	Test a range of amino acids with calcium imaging	Observed high responses to Arg > Lys > Ala > Ser	Pro is a weak Tas1R1/Tas1R3 agonist and did not elicit responses in tanycytes. No response when Arg applied on parenchyma	Fig. 1
B. Is the response enhanced by IMP?	Compare responses with and without IMP in medium	The presence of IMP immediately increased reponses to Arg	Time-matched control did not show an increase in responses	Fig. 2
C1. Is the response mediated by ATP?	Measure responses when P2 receptors are blocked	A combination of P2 antagonists blocked tanycyte responses to AAs	Each P2 antagonist alone had no effect; time-matched control showed no reduction in responses	Fig. 3; Supp. Fig. 1
C2. Do tancytes release ATP?	Measure ATP release directly with biosensors	ATP release was detected immediately after AA application	A 'null' biosensor not containing an enzyme; no response when Arg applied on parenchyma; a much smaller response when biosensor is placed in arcuate nucleus	Fig. 4
D. How is ATP released from tanycytes?	Block various large conductance channels	Panx1 antagonist reduced responses to Arg; Calhm1 antagonist reduced responses to Ala	Cx43 antagonist and time- matched control did not show a reduction in responses	Fig. 5
E. Is Tas1R1 expressed in tanycytes?	Image fixed brain slices from mice expressing GFP at the *Tas1R1* locus	GFP was observed in tanycytes, ependymal cells and neurons	No GFP in other areas of the brain	Fig. 6
F. Is Tas1R1 required for amino acid sensing?	Test Tas1R1-KO mice for AA responses using Ca imaging and biosensing	All mice: reduced responses to Lys; female mice: reduced responses to Arg; male mice: increased responses to Arg	WT mice (sex and age matched) as positive controls	Fig. 6
G. Are mGluRs required for tanycyte amino acid sensing?	Measure responses when mGluR1 or mGluR4 is blocked	mGluR4 antagonist MAP4 stopped responses to Ala and reduced responses to Lys	mGluR1 antagonist 4CPG had no effect; MAP4 did not affect Arg	Fig. 7
