# Supplementary material for: Amino acid sensing in hypothalamic tanycytes via umami taste receptors
Source: Mol Metab. 2017 Sep 14;6(11):1480–92. doi: 10.1016/j.molmet.2017.08.015 (PMC5681271; doi:10.1016/j.molmet.2017.08.015)
Supplement: Supplementary Figure 1 — The mechanism of ATP detection with microelectrode biosensors. (a) Enzymatic scheme of the ATP biosensor. The cascade of enzyme reactions produces H2O2 when ATP is present. Glycerol is added to the bathing medium prior to ATP measurements. H2O2 is oxidized on the electrode surface (polarized to 500 mV). (b) The Null sensor is an exact copy of the ATP biosensor without the enzymes required for ATP detection. It is therefore only sensitive to electrical disturbances and non-specific events, allowing exclusion of any noise or artefacts from the ATP biosensor recording [24]. [file mmc3.pdf]

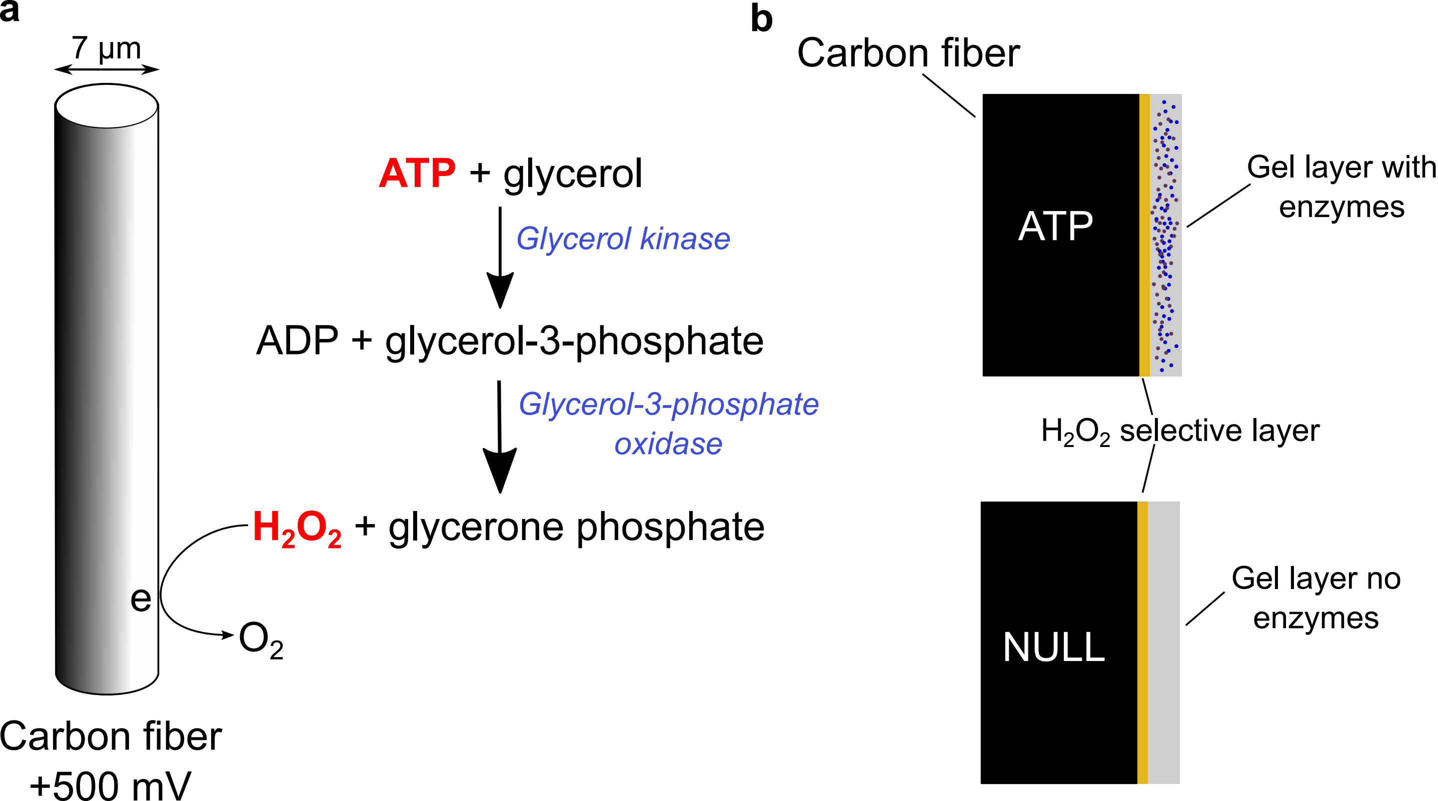

a
7 µm
ATP + glycerol
Glycerol kinase
ADP + glycerol-3-phosphate
Glycerol-3-phosphate oxidase
H_2O_2 + glycerone phosphate
e
O_2
Carbon fiber +500 mV
b
Carbon fiber
ATP
Gel layer with enzymes
H_2O_2 selective layer
NULL
Gel layer no enzymes
