# Supplementary figures and images for: Amino acid sensing in hypothalamic tanycytes via umami taste receptors
Source: Mol Metab. 2017 Sep 14;6(11):1480–92. doi: 10.1016/j.molmet.2017.08.015 (PMC5681271; doi:10.1016/j.molmet.2017.08.015)

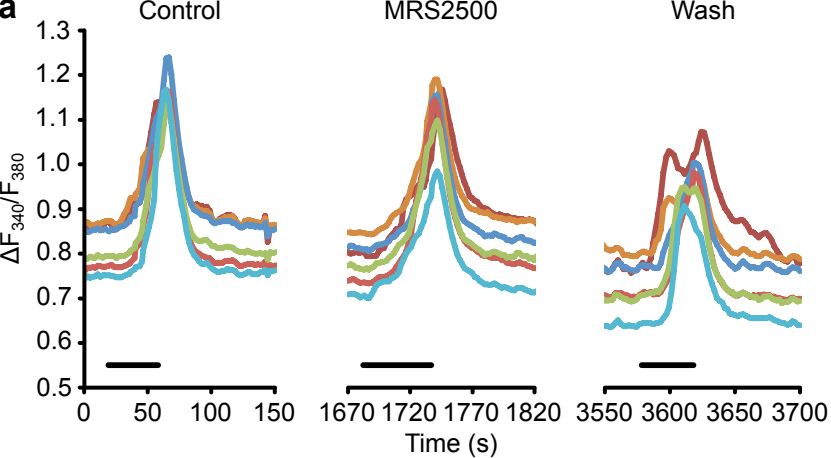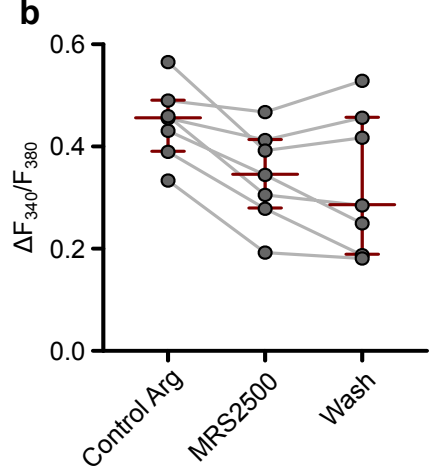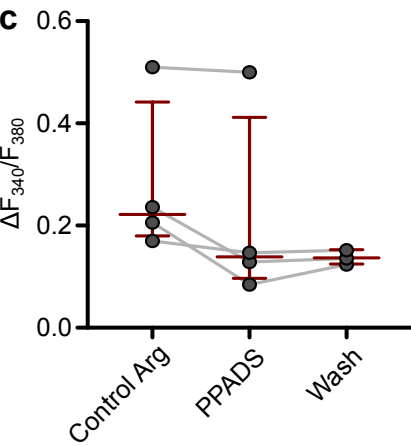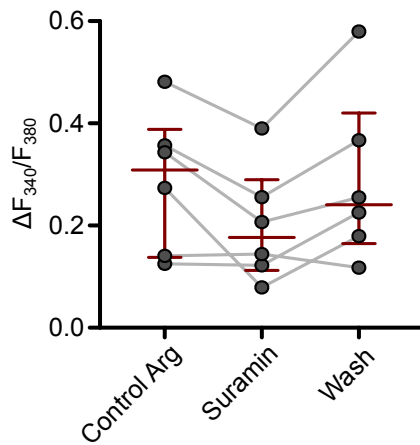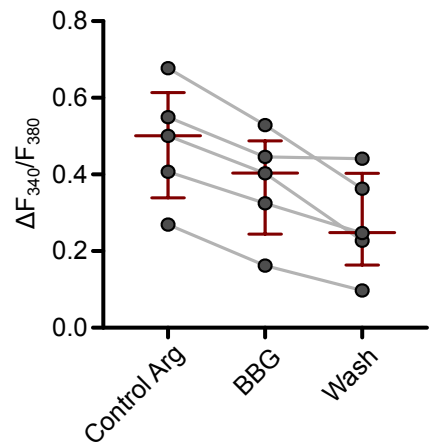

Supplement: Supplementary Figure 2 — l-arginine-evoked Ca2+signals in tanycytes are not blocked by application of single P2X or P2Y receptor antagonists. (a) Example ROI records of responses to arginine with and without 100 nM MRS2500. (b) Summary data for effect of MRS2500 on Arginine responses. (c) Summary data for effect of PPADS (30 μM), suramin (50 μM) or Brilliant Blue G (BBG, 10 μM) on arginine responses. [file mmc4.pdf]

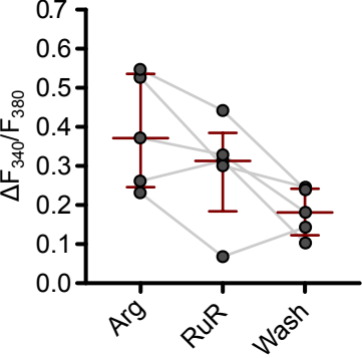

Supplement: Supplementary Figure 3 — Tanycyte responses tol-arginine are not mediated by CALHM1. The responses to l-arginine were unaffected by 50 μM Ruthenium Red. [file mmc5.pdf]

BL/6 Ala

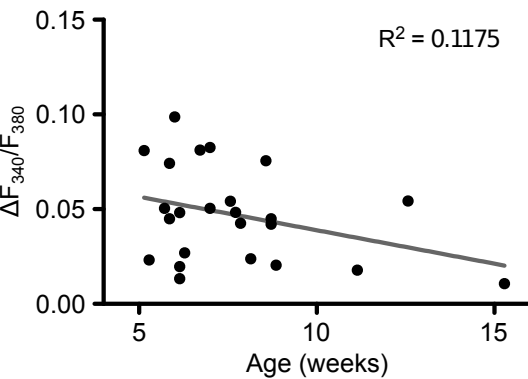

Tas1r1-KO Ala

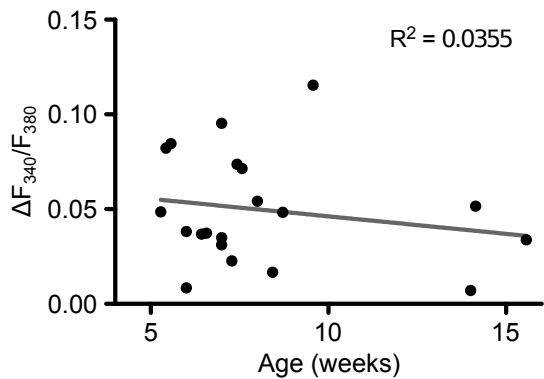

BL/6 Lys

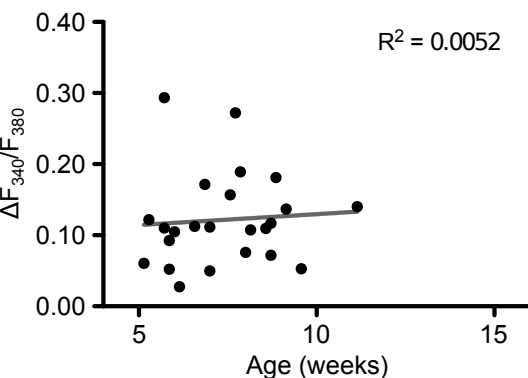

Tas1r1-KO Lys

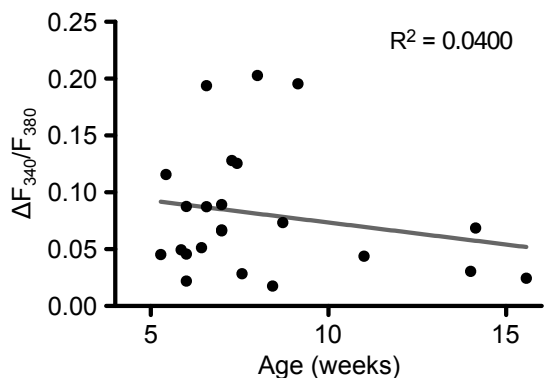

BL/6 Arg

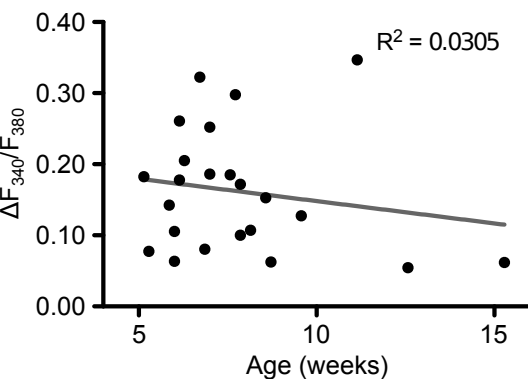

Tas1r1-KO Arg

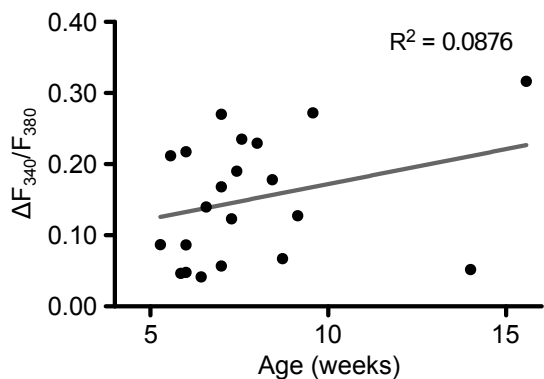

Supplement: Supplementary Figure 4 — Mouse tanycyte responses tol-amino acids do not change between the age of 5 and 16 weeks. In both wild type and Tas1r1-null mice used in our experiments, there was no correlation between the age of the animals and the amplitude of their responses to l-alanine, l-lysine or l-arginine. [file mmc6.pdf]
